# *Fasciola gigantica* excretory-secretory products (FgESPs) modulate the differentiation and immune functions of buffalo dendritic cells through a mechanism involving DNMT1 and TET1

**DOI:** 10.1186/s13071-020-04220-0

**Published:** 2020-07-17

**Authors:** Xuefang Mei, Wei Shi, Wenping Zhao, Honglin Luo, Yaoyao Zhang, Yurui Wang, Zhaoan Sheng, Dongying Wang, Xing-Quan Zhu, Weiyi Huang

**Affiliations:** 1 School of Animal Science and Technology, Guangxi University, Nanning, 530005 Guangxi Zhuang Autonomous Region People’s Republic of China; 2 School of Preclinical Medicine, Guangxi Medical University, Nanning, 530021 Guangxi Zhuang Autonomous Region People’s Republic of China; 3 Guangxi Key Laboratory for Aquatic Genetic Breeding and Healthy Aquaculture, Guangxi Institute of Fishery Sciences, Nanning, 530021 Guangxi Zhuang Autonomous Region People’s Republic of China; 4 State Key Laboratory of Veterinary Etiological Biology, Key Laboratory of Veterinary Parasitology of Gansu Province, Lanzhou Veterinary Research Institute, Chinese Academy of Agricultural Sciences, Lanzhou, 730046 Gansu People’s Republic of China; 5 Jiangsu Co-innovation Center for the Prevention and Control of Important Animal Infectious Diseases and Zoonoses, Yangzhou University College of Veterinary Medicine, Yangzhou, 225009 Jiangsu People’s Republic of China

**Keywords:** *Fasciola gigantica*, Excretory/secretory products, Dendritic cells, Differentiation, Immune functions

## Abstract

**Background:**

*Fasciola gigantica* infection threatens the health of both humans and animals in the world. The excretory/secretory products (ESPs) of this fluke has been reported to impair the activation and maturation of immune cells. We have previously shown the influence of *F. gigantica* ESPs (FgESPs) on the maturation of buffalo dendritic cells (DCs). However, the underlying mechanisms remain unclear. The objective of this study was to investigate the potency of FgESPs in shifting the differentiation and immune functions of buffalo DCs.

**Methods:**

Buffalo DCs were incubated with FgESPs directly or further co-cultured with lymphocytes in vitro. qRT-PCR was employed to determine the gene expression profile of DCs or the mixed cells, and an ELISA was used to measure cytokine levels in the supernatants. Hoechst and Giemsa staining assays, transmission electron microscopy, caspase-3/7 activity test and histone methylation test were performed to determine DC phenotyping, apoptosis and methylation. To investigate the mechanism involved with DNA methylation, a Co-IP assay and immunofluorescent staining assay were performed to observe if there was any direct interaction between FgESPs and DNMT1/TET1 in buffalo DCs, while RNAi technology was employed to knockdown DNMT1 and TET1 in order to evaluate any different influence of FgESPs on DCs when these genes were absent.

**Results:**

qRT-PCR and ELISA data together demonstrated the upregulation of DC2 and Th2/Treg markers in DCs alone and DCs with a mixed lymphocyte reaction (MLR), suggesting a bias of DC2 that potentially directed Th2 differentiation in vitro. DC apoptosis was also found and evidenced morphologically and biochemically, which might be a source of tolerogenic DCs that led to Treg differentiation. In addition, FgESPs induced methylation level changes of histones H3K4 and H3K9, which correlate with DNA methylation. Co-IP and immunofluorescent subcellular localization assays showed no direct interaction between the FgESPs and DNMT1/TET1 in buffalo DCs. The productions of IL-6 and IL-12 were found separately altered by the knockdown of DNMT1 and TET1 in DCs after FgESPs treatment.

**Conclusions:**

FgESPs may induce the DC2 phenotype or the apoptosis of buffalo DCs to induce the downstream Th2/Treg response of T cells, possibly through a DNMT1- or TET1-dependent manner(s).
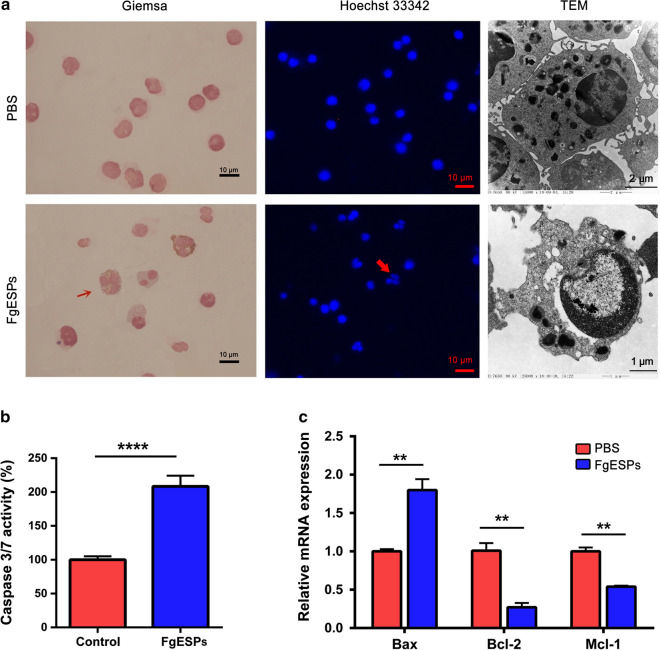

## Background

Fascioliasis caused by *Fasciola hepatica* in temperate regions and *Fasciola gigantica* in tropical regions has been considered an important but neglected zoonosis with an increasing number of people and livestock animals infected around the world [1]. Both *F. hepatica* and *F. gigantica* are able to induce a suppressive immune response to offset the eliminatory effects of the host during the infection [2–4], where the dynamic changing profile of pro-inflammatory and anti-inflammatory cytokines greatly influences the progression of the disease [5, 6]. Numerous previous studies have revealed that these flukes can produce various molecules to help evading host immune surveillance and clearance through impacting activation, development and functions of immune cells, including dendritic cells (DCs) [7–9], macrophages [10], mast cells [11, 12], and T cells [2].

Among the immune cells, DCs as the major well-known antigen-presenting cells (APCs) linking innate and adaptive immunity, play a key role in the initiation of protective pro-inflammatory as well as tolerogenic immune responses through continuously capturing and responding to the pathogenic antigens from microenvironment [13]. Helminths and helminth-derived molecules condition DCs to generate a non-classical maturation during infection, and prime a protective Th2 or Treg response which cause less damage to either the parasite or the host [14, 15]. These multicellular flukes, unlike protozoans and microorganisms, release excretory-secretory products (ESPs) to influence the host immune system as a useful strategy for survival. It has been reported that the infection with *F. hepatica* could be recognized by multiple pattern recognition receptors (PRRs) including toll-like receptors (TLRs) on the APCs, which has not been fully elucidated. The ESPs of *F. hepatica* (FhESPs) suppress DC maturation and functions [7–9], inhibit TLR4 activation, and suppress the production of inflammatory cytokines [7, 10, 16]. Because of this, FhESPs impairs pro-inflammatory immune response through suppressing the classical maturation of DCs. *Fasciola gigantica* and its products are known to elicit a modest Th2 response to attenuate the harmful Th1-type and Th17-type pro-inflammatory responses during the early stage of infection, but evoke a mixed type of Th1 and Th2 cytokine environment when the infection progresses to a chronic stage [5, 17, 18]. However, impacts of *F. gigantica* and its ESPs on DC differentiation and immune functions still remain unclear.

Buffaloes are important economic livestock in many developing countries in Asia and Africa, and also the most common natural host for *F. gigantica*. The infection with *F. gigantica* in buffaloes has caused huge economic losses to the buffalo industry in these areas. However, little is known about the potential role of buffalo DCs in infection especially parasitosis. Our latest study demonstrated that *F. gigantica* ESPs (FgESPs) could disrupt the maturation and functions of buffalo DCs by altering the status of DNA (hydroxy)methylation [19]. As an indispensable continuity study, here, we first designed and conducted a series of experiments to investigate the major effects of FgESPs on the differentiation of water buffalo peripheral blood mononuclear cell (PBMC)-derived DCs in vitro, with the aim to provide a comprehensive understanding of the interaction between FgESPs and buffalo DCs. Moreover, it has been reported that among several genes involving DNA (hydroxy)methylation, DNA methyltransferase (DNMT) 1 and ten-eleven translocation (TET) 1 play some key roles. A study on DCs has shown that the miR-148a/DNMT1/SOCS1 regulatory axis, as a negative regulator for TLR, triggered DC maturation [20]. Reducing the expression of the DNMT1 gene in cattle embryos by RNA interference (RNAi) could induce DNA demethylation [21]. Similarly, silencing the TET1 gene by RNAi reduces the level of 5-hydroxymethylcytosine (5-hmC) but increases 5-methylcytosine (5-mC) in the promoter region of DNA (hydroxy)methylation related genes [22–24], due to the regulatory effects of TET1 on 5-mC and 5-hmC, and following the dynamic balancing of DNA (hydroxy)methylation. So, as an extension of evaluating the effects of FgESPs to buffalo DC state, we then investigated the roles of DNMT1 and TET1 involving the interaction between FgESPs and DCs based on the approaches of co-immunoprecipitation (Co-IP) and RNAi assay. This study provided some new knowledge which filled up many gaps in the better understanding of the infection mechanism of *F. gigantica*.

## Methods

### Animals and FgESPs preparation

Three-year-old clinically healthy female water buffaloes (Guangxi breed, swamp type) were purchased from a local breeder in Nanning, Guangxi, China. Antibiotics and anthelmintics were given by following the manufacturer’s instructions, as a routine procedure to avoid any possible pre-existing infections. After 4 weeks of drug-withdrawal, the animals were confirmed as free of common pathogens by negative fecal examination and negative blood smear examination.

FgESPs was prepared as described previously with slight modifications [9]. Briefly, adult worms were chosen and cultured in PBS containing glucose (1 worm/2 ml) at 37 °C for another 2 h. The supernatant was collected, centrifuged at 12,000× *g* for 30 min at 4 °C and filtered through a 0.22 μm Millipore filter. The collection was then processed by vacuum freeze-drying. All procedures above strictly followed the standards of aseptic operation to avoid any possible contamination. The freeze-dried powder of FgESPs was aliquoted and stored at − 80 °C until use.

To prepare polyclonal antibody (pAb)-containing serum, two of 6-month-old clinically healthy female New Zealand rabbits were experimentally immunized with FgESPs combined with routine adjuvant *via* intramuscular (i.m.) injection for 4 times. Blood was drained and collected from the rabbit when the specific Ab titer reached ~ 64,000. Serum containing all Abs was separated by centrifugation, then purified by affinity chromatography using a Protein A column. Brief scheme of the procedures for generating FgESPs-specific pAb was shown as Additional file 2: Figure S1a. The characteristic band profile pattern of FgESPs was determined by SDS-PAGE and visualized by Coomassie blue staining. The specificity of pAb to FgESPs was tested by western-blot assay (Additional file 2: Figure S1b, c).

### Preparation and culture of DCs and lymphocytes

PBMCs and PBMC-derived DCs were generated from the carotid venous blood of one healthy buffalo, and identified by following the procedures described in our previous study [19]. The non-adherent cells (containing lymphocytes) were collected immediately after the above step of PBMC adherence. The lymphocytes were further separated from non-adherent cells using the nylon-wool column method [25]. Cells were washed, resuspended in complete RPMI-1640 culture medium (Gibco, Grand Island, NY, USA) containing 10% fetal bovine serum (Gibco), 1% L-Glutamax (Gibco), and 1% PSN (Sigma-Aldrich, St. Louis, MO, USA), and seeded into a Petri dish to allow adherence for 2 h at 37 °C in a 5% CO_2_ incubator. Lymphocytes with high viability assessed by trypan blue exclusion staining, were allowed to be used for the following stimulation and tests.

### In vitro treatments of DCs

To investigate the direct reaction of buffalo DCs to the stimulation of FgESPs in vitro, 1 × 10^6^ of the generated buffalo DCs per well with three replicate wells as treatment were incubated with 200 μg/ml of FgESPs (optimum dosage according to our unreported pre-screening test previously) diluted in phosphate-buffered solution (PBS) for 48 h, whereas cells treated with an equivalent volume of PBS served as the control. The treated DCs were collected for further gene expression analysis of interferon (IFN)-γ, cluster of differentiation (CD) 86, interleukin (IL)-4, IL-10, IL-13, complement component 1 q subcomponent A chain (C1QA), stabilin-1 (STAB1), Mcl-1, Bcl-2 and Bax, which would provide information for the status of the phenotype and apoptosis, employing the quantitative reverse transcription polymerase chain reaction (qRT-PCR). In another set of experiment, an allogeneic mixed lymphocyte reaction (MLR) model was employed as a further and more functional discussion for FgESPs on DCs. The DCs were pre-treated with 200 μg/ml FgESPs or equal volumes of PBS (as a blank) for 48 h as above. Then, pre-treated DCs (2 × 10^5^ cells/well) were cultured in flat-bottom 24-well plates with 1 × 10^6^ buffalo lymphocytes for 4 days. The mixed cells were collected and processed for qRT-PCR analysis of the relative mRNA levels of T cell markers and cytokines associated with T cell differentiation and immunological functions, including T-bet, GATA binding protein 3 (GATA3), Foxp3, IFN-γ, IL-4, IL-10, IL-13 and transforming growth factor beta (TGF-β), by qRT-PCR. The cell culture supernatants were collected for cytokine excretion determination using an enzyme-linked immunosorbent assay (ELISA).

### Hoechst 33342 and Giemsa staining assays

Bisbenzimide (Hoechst 33342; Sigma-Aldrich) was used here as a live cell DNA stain probe to highlight the formation of apoptotic bodies during cell apoptosis. The buffalo DCs treated with or without FgESPs for 48 h were harvested and transferred into a round-bottom Eppendorf tube in triplicates. Hoechst 33342 was added to the tube according to the manufacturer’s instructions, and incubated at room temperature in the absence of light for 20–30 min. After centrifugation at 1500× *rpm* and two washes in cold PBS, cells were made into smear slides, and observed under a Nikon 80i fluorescence microscope (Nikon, Milton Keynes, UK) with a digital camera attached to the computer. The apoptotic cells were then quantified by the mean percentage of Hoechst-positive nuclei (blue color) per optical field from at least 10 fields. Giemsa staining was also performed to display the microscopic structure of the cells. The cells were collected, washed, and fixed to the slide with 4% paraformaldehyde (PFA) solution. After air-drying for a few minutes, Giemsa stain solution (10× stock solution; Solarbio, Beijing, China) diluted in PBS (v:v = 1:9) was added dropwise to the stained cells. The slide was dehydrated, vitrified by xylol and mounted in neutral balsam. The microscopic structure of the stained cells was observed and photographed under a light microscope.

### Ultrastructural characterization

The ultrastructural characteristics of the DCs were observed under a transmission electron microscope (TEM). After treatment with or without FgESPs for 48 h, the generated buffalo DCs were fixed in 3% glutaraldehyde, followed by treatment with 1% osmium tetroxide at 4 °C overnight. Then, they were dehydrated in a graded ethanol series (50, 70, 80, 90, 95 and 100%), embedded in epon resin, sectioned on a Leica ultramicrotome (EM-UC7; Leica, Wetzlar, Germany) and stained with uranyl acetate and lead citrate. The intracellular structural changes of the samples were then observed under TEM (S-3400N; Hitachi, Tokyo, Japan).

### Caspase-3/7 activity assay

The buffalo DCs treated with or without FgESPs were harvested after appropriate treatments and placed into a 96-well flat-bottom microplate in triplicate (5 × 10^3^ cells in 100 μl medium per well). Then, 100 μl of Caspase-Glo 3/7 Reagent (Promega, Madison, WI, USA) was added to each well of the plate, followed by 1–2 min mixing on a plate shaker. The plate was incubated at room temperature for 3 h. Luminescence at 485Ex/527Em was measured in a microplate reader (PerkinElmer, Waltham, MA, USA). The apoptosis index was calculated as the ratio of cells with apoptotic nuclei to the total number of cells in each group.

### qRT-PCR

Gene expression was determined by qRT-PCR. Total cellular RNA was extracted using the RNAiso Plus Kit (TaKaRa, Beijing, China) following the manufacturerʼs protocol. The quality and quantity of the isolated RNA was subsequently analyzed using a Nanodrop-2000C (Thermo Fisher Scientific, Wilmington, DE, USA). The complete complementary DNA (cDNA) was reverse transcribed from 500 ng of total cellular RNA using the PrimerScript™ RT Reagent Kit (TaKaRa) according to the manufacturer’s recommendations. The mRNA expression of genes was examined by qRT-PCR using ChamQ SYBR Color qPCR Master Mix (Vazyme Biotech, Nanjing, China) and a CFX96 real-time PCR instrument (Bio-Rad, Hercules, CA, USA). The sequences of each primer set for genes of interest are listed in Additional file 1: Table S1. Analysis of relative mRNA expression of target genes was performed using the 2^*−*ΔΔCq^ method [26]. Normalization was accomplished using bubaline glyceraldehyde-3-phosphate dehydrogenase (GAPDH) as the reference housekeeping gene [27].

### ELISA

For the excretion levels of cytokines, IFN-γ, IL-4, IL-6, IL-10, IL-12, IL-13, tumor necrosis factor alpha (TNF-α) and TGF-β were detected in culture supernatants of the co-culture cells by a commercial ELISA kit (Cloud-Clone, Wuhan, China), according to the manufacturer’s instructions.

### Global histone methylation assay

Histone extraction and the detection of Lys4 of histone H3 (H3K4)/H3K9/H3K27 methylation were performed using the EpiQuik™ global histone H3K4/H3K9/H3K27 methylation assay kits (Epigentek, Farmingdale, NY, USA), according to the manufacturer’s instructions.

### Bioinformatic prediction for buffalo DNMT1 and TET1

Given the dearth of experimentally validated information for buffalo DNMT1 and TET1 proteins, we aimed to predict the possible subcellular localization for these two proteins by using bioinformatics tools. Amino-acid sequences of DNMT1 or TET1 from different species were downloaded from the NCBI GenBank amino-acid database, and their GenBank accession numbers are listed as below: for DNMT1, water buffalo (*Bubalus bubalis*) XP_025149334.1, cattle (*Bos taurus*) NP_872592.2, pig (*Sus scrofa*) NP_001027526.1, human (*Homo sapiens*) NP_001124295.1, monkey (*Macaca mulatta*) XP_028695109.1, gorilla (*Pongo abelii*) XP_024093741.1, horse (*Equus przewalskii*) XP_008529102.1, mouse (*Mus musculus*) XP_006510051.1, rat (*Rattus norvegicus*) NP_445806.3; for TET1, water buffalo XP_025140415.1, cattle XP_015316519.1, pig XP_013845862.1, human NP_085128.2, monkey XP_015002985.1, gorilla XP_002820931.1, horse XP_008528402.1, mouse NP_001240786.1, rat XP_008773174.1. Phylogenetic tree among common mammal species was constructed by MEGA 6.0 software. Prediction of protein transmembrane (TM) domain used TMHMM Serve v2.0 online software (http://www.cbs.dtu.dk/services/TMHMM/). Signal peptide (SP) domain was analyzed by using SignalP v4.1 online software (http://www.cbs.dtu.dk/services/SignalP-4.1/). Nuclear localization was predicted by ProtComp v9.0 online software (http://linux1.softberry.com/berry.phtml?topic=protcomppl&group=programs&subgroup=proloc) and PSORT II (https://psort.hgc.jp/form2.html).

### Co-IP assay

The total protein was extracted from FgESPs-treated buffalo DCs using RIPA Lysis Buffer (Beyotime, Shanghai, China) containing 1% phenylmethanesulfonyl fluoride (PMSF as a protease inhibitor, v/v; Beyotime) on ice. The soluble protein was separated from the lysate of cellular debris by centrifugation at 10,000× *g* for 30 min at 4 °C, and the supernatant was transferred to a new tube. For co-immunoprecipitation of the intracellular DNMT1 and TET1 targets bounding to any component of FgESPs intake in DCs, 0.75 mg of whole cell extracts for buffalo DCs were incubated with anti-TET1 (Santa Cruz, CA, USA), anti-DNMT1 (Abcam, Cambridge, UK) or normal mouse IgG (Santa Cruz) in PBS supplemented with 10% glycerin (v/v) and 1% protease inhibitors (v/v) at 4 °C overnight, respectively. Protein G was added to the antibody-antigen complex at 20 μl per sample and incubated at 4 °C for 4 h, then 50 μl of HCl (pH = 2.5) was added and incubated at 4 °C for 10 min. After sufficiently washing the beads 5 times, the bound proteins were removed from the beads. Prior to the following analyses, the proteins were quantitated by a Bicinchoninic Acid (BCA)-based assay, using a BCA Protein Assay Kit (Beyotime). The proteins were adjusted to an equivalent concentration by adding an appropriate volume of PBS. Then, 10 μl of the sample was used for confirmation of the binding of FgESPs-specific components to DNMT1 and TET1 by western blot using a laboratory-made rabbit polyclonal antibody against the whole antigen of FgESPs (Additional file 2: Figure S1). The negative control was conducted in exactly the same way, only without FgESPs incubation.

### Fluorescent subcellular localization of FgESPs

The total protein of FgESPs was pre-stained using a fluorescent Cy3 NHS ester (ApexBio Technology, Houston, TX, USA). The buffalo DCs were incubated in the presence of 200 μg/ml of the Cy3-conjugated FgESPs for 24 h at 37 °C in 5% CO_2_ incubator. The washed cells (10^5^ cells/ml) were collected and fixed with 4% PFA on a poly-l-lysine-coated glass slide. After air-drying, the cells were incubated with the lipophilic fluorescent dye DiO (final concentration 5 μM; Beyotime) at 4 °C for the cell membranous staining. After incubation for 30 min in the dark, the cell slide was washed, mounted in an antifade solution containing the nuclear dye DAPI (Solarbio), and covered with a coverslip. The protein subcellular localization was then determined by observing the staining patterns on an inverted Zeiss microscope (Axio Imager Z2; Zeiss, Jena, Germany) with a 100× oil objective lens. Digital images were captured using the proprietary Zeiss software package.

### Western blotting analysis

The soluble proteins were denatured by adding 4× SDS-PAGE Loading Buffer (TaKaRa) and heating at 95 °C for 10 min. Samples were separated by sodium dodecyl sulfate polyacrylamide gel electrophoresis (SDS-PAGE) in SDS-containing polyacrylamide gels (12%) and transferred to polyvinylidene fluoride (PVDF) membranes (Solarbio). Membranes were then blocked for 60 min with PBS containing 0.1% Tween-20 and 5% skimmed milk powder. Anti-mouse DNMT1 mAb (Abcam), anti-human TET1 mAb (Santa Cruz) and anti-mouse β-actin mAb (Cloud-Clone) were used as the primary antibodies for the following Western blotting detection, whereas goat anti-mouse IgG-HRP (Cloud-Clone) was used as the secondary antibody. Grayscale values of each band were measured with an ImageJ software. Grayscale ratio equals to the value of target protein divided by the value of β-actin, which reflects the relative expression of target proteins shown under the corresponding bands.

### RNAi for TET1and DNMT1

RNAi technology was used to knockdown the gene expression of DNMT1 or TET1 in the generated buffalo DCs in vitro. Small interfering RNA (siRNA) duplexes against the coding sequences of buffalo DNMT1 and TET1 (GenBank: XM_006044441.1 and XM_006070286.1, respectively), were designed and synthesized commercially from GenePharma Co., Ltd (Shanghai, China). A BLAST search was conducted at the National Center for Biotechnology Information (NCBI) against the DNMT1 and TET1 genes to confirm the target specificity of designed siRNAs in prior. The following sequences were generated after optimization: DNMT1 forward (5′-GAC UCA GAA GUC AAA CCA ATT-3′) and reverse (5′-UUG GUU UGA CUU CUG AGU CTT-3′); TET1 forward (5′-GGA GCA GCA CGA AUG AAU UTT-3′) and reverse (5′-AAU UCA UUC GUG CUG CUC CTT-3′); negative control (mock siRNA) forward (5′-UUC UCC GAA CGU GUC ACG UTT-3′) and reverse (5′-ACG UGA CAC GUU CGG AGA ATT-3′). The transfection of siRNAs into DCs was performed using Lipofectamin 3000 Reagent (Invitrogen, Carlsbad, CA, USA) following the manufacturer’s instructions. The detection of interference efficiency is shown in Additional file 3: Figure S2.

In order to test if the transfection of mock siRNA would cause any changes to the normal DCs, we performed a comparative qRT-PCR analysis on genes of common DC markers (listed in Additional file 1: Table S1) for cDNA of the transfected DCs and non-transfected normal DCs, and measured the production profile cytokines IL6, IL-10, IL-12 and TNF-α in the cell culture supernatant using commercial ELISA kits (Cloud-Clone) according to the manufacturer’s instructions. Then, the DCs with or without gene-knockdown, were respectively transferred and cultured in a 24-well flat cell culture plate with a density of 1 × 10^6^ cells per well. Cells received treatment for 48 h with 200 μg/ml of FgESPs diluted in PBS, and equivalent volumes of PBS as a negative control, in triplicate. To evaluate the effects of FgESPs on the DCs with or without gene-knockdown, the cells were collected and detection of relative mRNA expression of CD1a, CD40, CD80, CD83, CD86 and major histocompatibility complex (MHC) II by qRT-PCR was performed. The secretion of IL-6, IL-10, IL-12 and TNF-α were detected in culture supernatants using the commercial ELISA kit (Cloud-Clone) according to the manufacturer’s instructions.

### Statistical analysis

The statistical analyses (including graphs) were performed using GraphPad Prism v7.02 (GraphPad Software, San Diego, CA, USA). All data represent the mean ± standard error (SE) of triplicate measurements. A paired t-test was performed followed by a two-tailed *post-hoc* test to evaluate the difference in changes between FgESPs treatment and PBS control in all experiments, with the exception of data from RNAi for DNMT1/TET1. For the statistical analysis of RNAi part, one-way analysis of variance (ANOVA) was performed for each biomarker or cytokine to identify the effect of siRNA(s) to DCs. The level of significance for all analyses was determined with a confidence interval > 95% (*P < 0.05, **P < 0.01, ***P < 0.001, ****P < 0.0001).

## Results

### FgESPs possibly induces a DC2 phenotype that activates Th2 and Treg cell differentiation in vitro

**Fig. 1:**
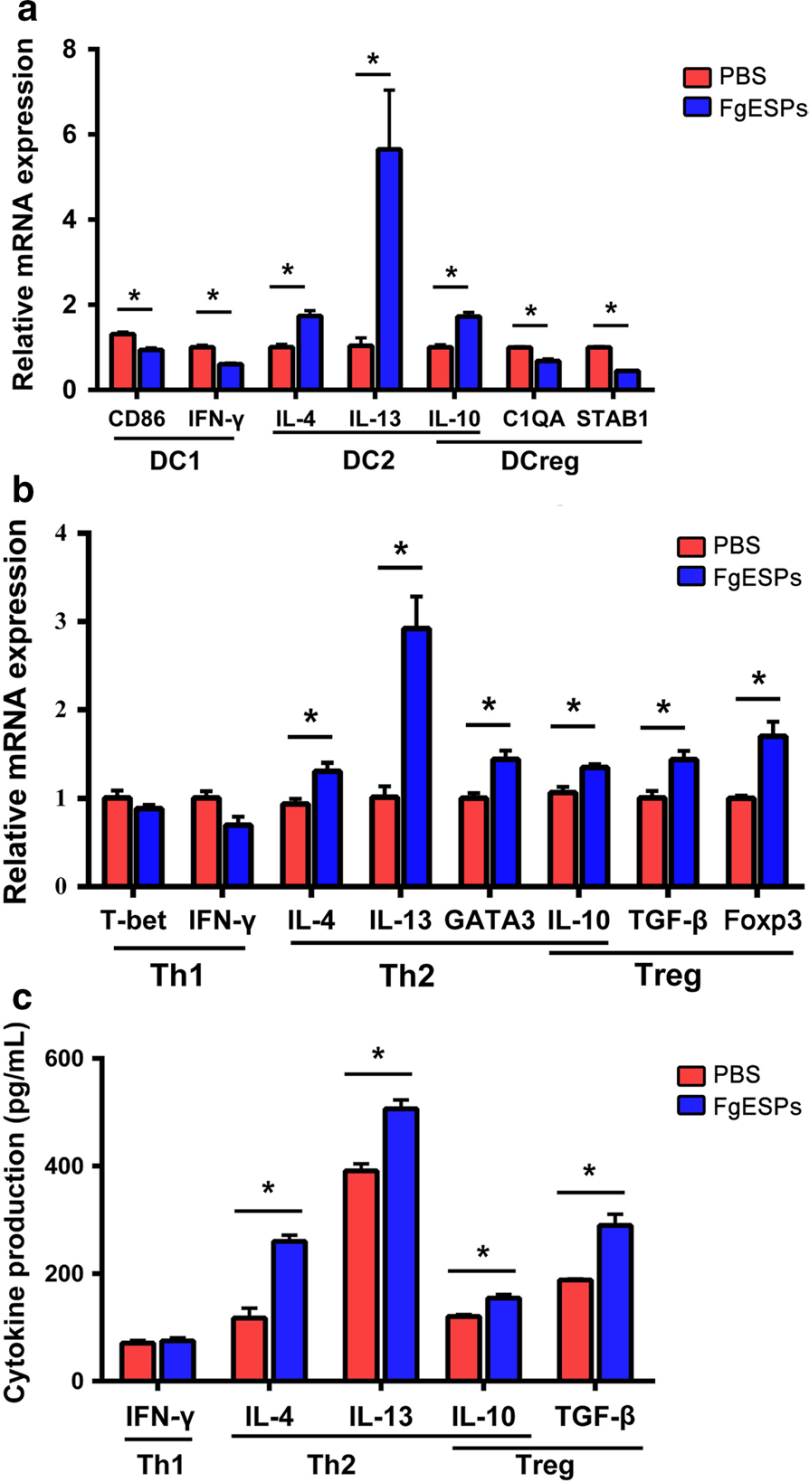
FgESPs induce differentiation of buffalo DCs and the cascaded T cells in vitro. **a** The mRNA levels of the most representative phenotype-associated markers for DC1, DC2 and DCreg cluster in buffalo DCs after incubation with FgESPs or PBS for 48 h. **b** The mRNA levels of the most representative cytokines and transcription factors for Th1, Th2 and Treg in the mixture of buffalo DCs and lymphocytes after the MLR in the presence of FgESPs or PBS for 48 h. The data is represented as the relative mRNA expression level, calculated by the 2^*−*ΔΔCq^ method. **c** Production of the most representative cytokines in co-culture supernatants of buffalo DCs and lymphocytes. Representative histogram from three independent experiments are shown. All data are presented as the mean ± standard error (SE) of triplicate measurements. *P < 0.05

In order to determine the direction of DC differentiation induced by FgESPs, DCs were incubated with FgESPs in vitro for 48 h, while PBS served as controls. The results showed that the mRNA levels of surface molecular markers and cytokine of DC1 (CD86, IFN-γ) and DCreg (C1QA, STAB1) were significantly lower than those in PBS-treated DCs (*t*_(2)_ = 9.888, *P* = 0.0101 for CD86; *t*_(2)_ = 5.464, *P* = 0.0319 for IFN-γ; *t*_(2)_ = 8.350, *P* = 0.0140 for C1QA; *t*_(2)_ = 9.890, *P* = 0.0101 for STAB1), while DC2 (IL-4, IL-10 and IL-13) were significantly higher (*t*_(2)_ = 7.216, *P* = 0.0187 for IL-4; *t*_(2)_ = 4.609, *P* = 0.0440 for IL-10; *t*_(2)_ = 4.363, *P* = 0.0487 for IL-13) (Fig. 1a).

To further confirm the direction of FgESPs-induced DC differentiation, we used a MLR system to determine the type of T cell subsets within lymphocytes mediated by differentiated DCs. The results showed that the gene expressions of GATA3, IL-4, IL-13 (Th2), TGF-β, Foxp3 (regulatory) and IL-10 (Th2/regulatory) were all significantly increased in the mixture of FgESPs-treated DCs and lymphocytes, rather than mixture of PBS-treated DCs and lymphocytes (*t*_(2)_ = 5.422, *P* = 0.0324 for GATA3; *t*_(2)_ = 5.410, *P* = 0.0325 for IL-4; *t*_(2)_ = 4.602, *P* = 0.0441 for IL-10; *t*_(2)_ = 7.560, *P* = 0.0170 for IL-13; *t*_(2)_ = 4.386, *P* = 0.0483 for TGF-β; *t*_(2)_ = 7.940, *P* = 0.0155 for Foxp3) (Fig. 1b). The secretion levels of Th2 cytokines IL-4, IL-10, IL-13 and regulatory cytokines TGF-β, IL-10 were significantly increased (*t*_(2)_ = 8.868, *P* = 0.0125 for IL-4; *t*_(2)_ = 4.474, *P* = 0.0465 for IL-10; *t*_(2)_ = 5.873, *P* = 0.0278 for IL-13; *t*_(2)_ = 4.865, *P* = 0.0397 for TGF-β) (Fig. 1c). Based on these data, we therefore speculate that FgESPs may induce DCs to differentiate into DC2, and possibly direct Th2 cells and Treg differentiation in vitro.

### FgESPs induces DC apoptosis in vitro

**Fig. 2:**
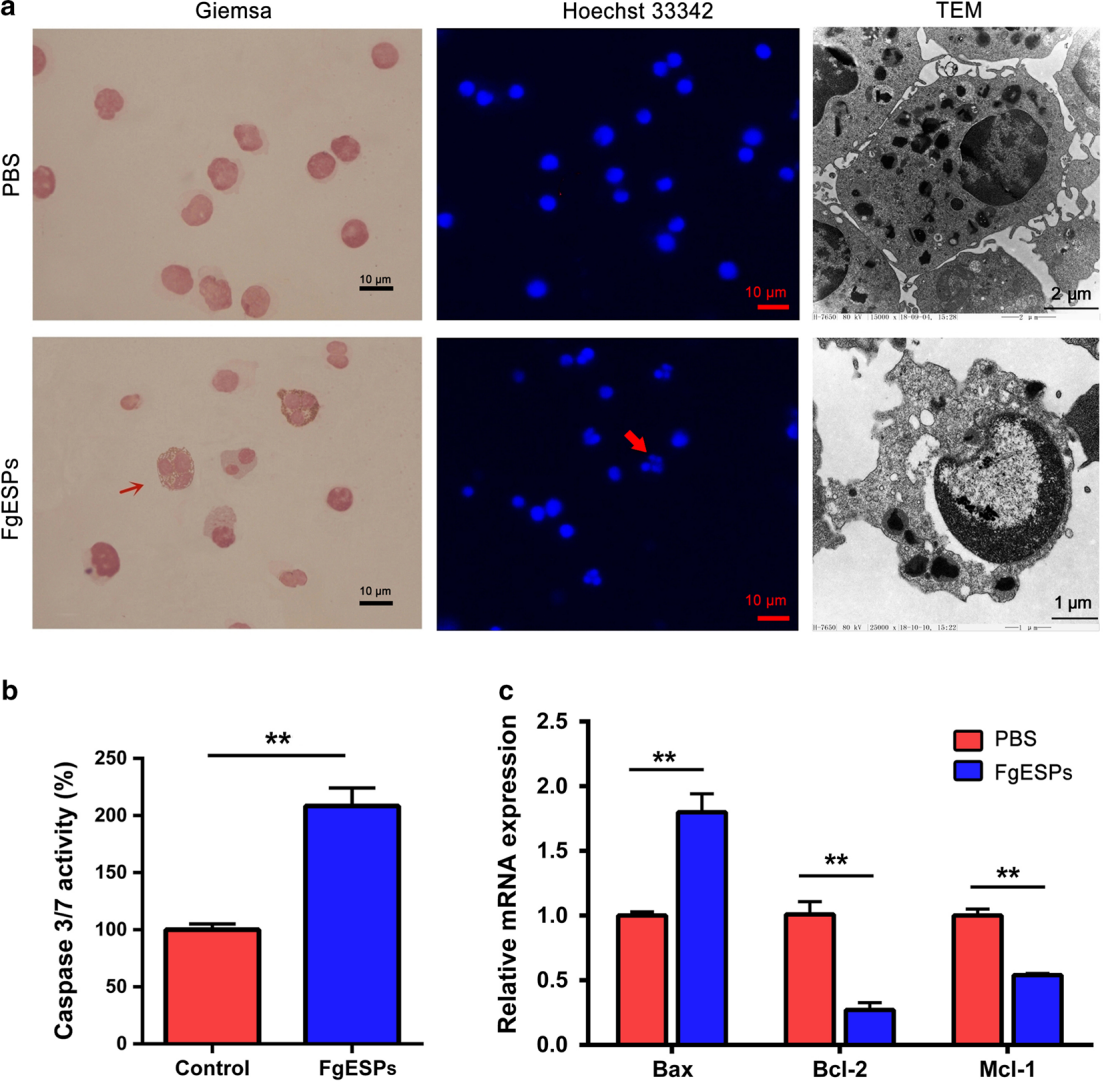
FgESPs induce apoptosis of buffalo DCs in vitro. **a** Buffalo DCs occurred morphological structure changes as representative of apoptotic features after the treatment of FgESPs for 48 h. **b** Caspase-3/7 activity was significantly increased in buffalo DCs after incubation with FgESPs. **c** The mRNA expression level of pro-apoptotic marker Bax was significantly upregulated, while the anti-apoptotic genes Bcl-2 and Mcl-1 were both downregulated in the FgESPs-treated DCs. The data are represented as the relative mRNA expression level, calculated by 2^*−*ΔΔCq^ method. Representative histograms from three independent experiments are shown. All data are presented as the mean ± standard error (SE) of triplicate measurements. **P < 0.01

The observation of Giemsa staining and TEM imaging showed that rather than the PBS-treated DCs, typical apoptotic features were present in DCs which were incubated with FgESPs, i.e. the numbers of lysosomes and phagosome-like balls were increased; and most characteristically, karyopyknosis (shrunken and dark nuclei) and karyorrhexis (nuclei fragmentation) were observed. By using Hoechst 33342 fluorescent staining, fragmented nuclei were found in a fair number (42 ± 4%) of DCs which were incubated with FgESPs but not in untreated DCs; while the fluorescent nuclear chromatin showed an even distribution, with a round or oval shape, without visible karyopyknosis in the control DCs (Fig. 2a). Caspase-3/7 measurement demonstrated the activity of caspase-3/7 in DCs treated with FgESPs was significantly higher than that of the control (*t*_(3)_ = 8.817, *P* = 0.0031) (Fig. 2b). The mRNA levels anti-apoptotic gene Mcl-1 and Bcl-2 was significantly lower in FgESPs-treated DCs (*t*_(2)_ = 13.44, *P* = 0.0055 for Mcl-1; *t*_(2)_ = 10.90, *P* = 0.0083 for Bcl-2), while the expression of the pro-apoptotic gene Bax was significantly increased (*t*_(2)_ = 10.20, *P* = 0.0095) (Fig. 2c). These data suggest that FgESPs induces DC apoptosis in vitro.

### FgESPs alter histone methylation of DCs in vitro

**Fig. 3:**
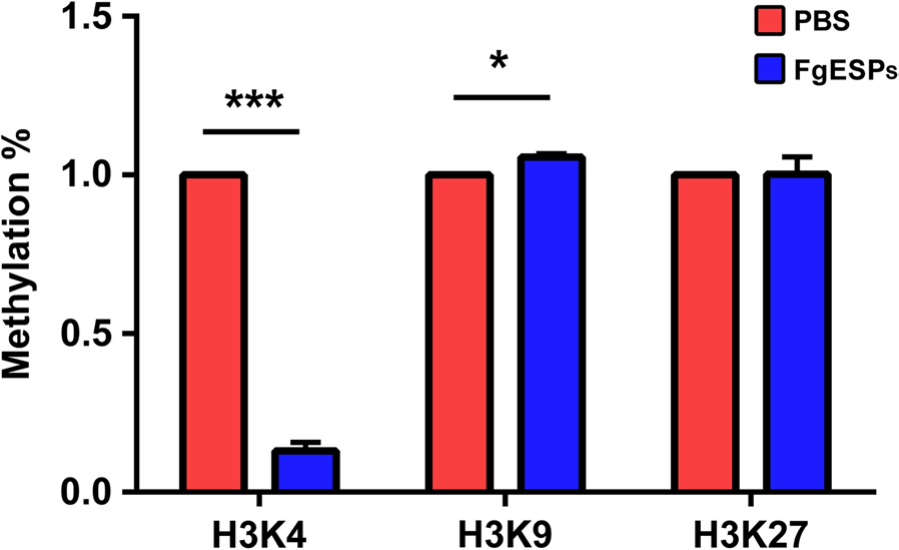
Effect of FgESPs on histone methylation levels in buffalo DCs. Calculation of the percentage of methylation level (‘Methylation %’ as shown on y-axis) was performed by following the manufacturer’s instructions. Representative histogram from three independent experiments are shown. All data are presented as the mean ± standard error (SE) of triplicate measurements. *P < 0.05, ***P < 0.001

As shown in Fig. 3, the result of the histone methylation test demonstrated that FgESPs treatment significantly reduced the H3K4 methylation level (*t*_(2)_ = 31.75, *P* = 0.0010), but increased the H3K9 methylation level (*t*_(2)_ = 5.179, *P* = 0.0353). However, the level of H3K27 methylation was found almost unchanged in FgESPs-treated DCs rather than the controls (*t*_(2)_ = 3.742, *P* = 0.0646).

### FgESPs do not directly interact with DNMT1 or TET1 in DCs

**Fig. 4:**
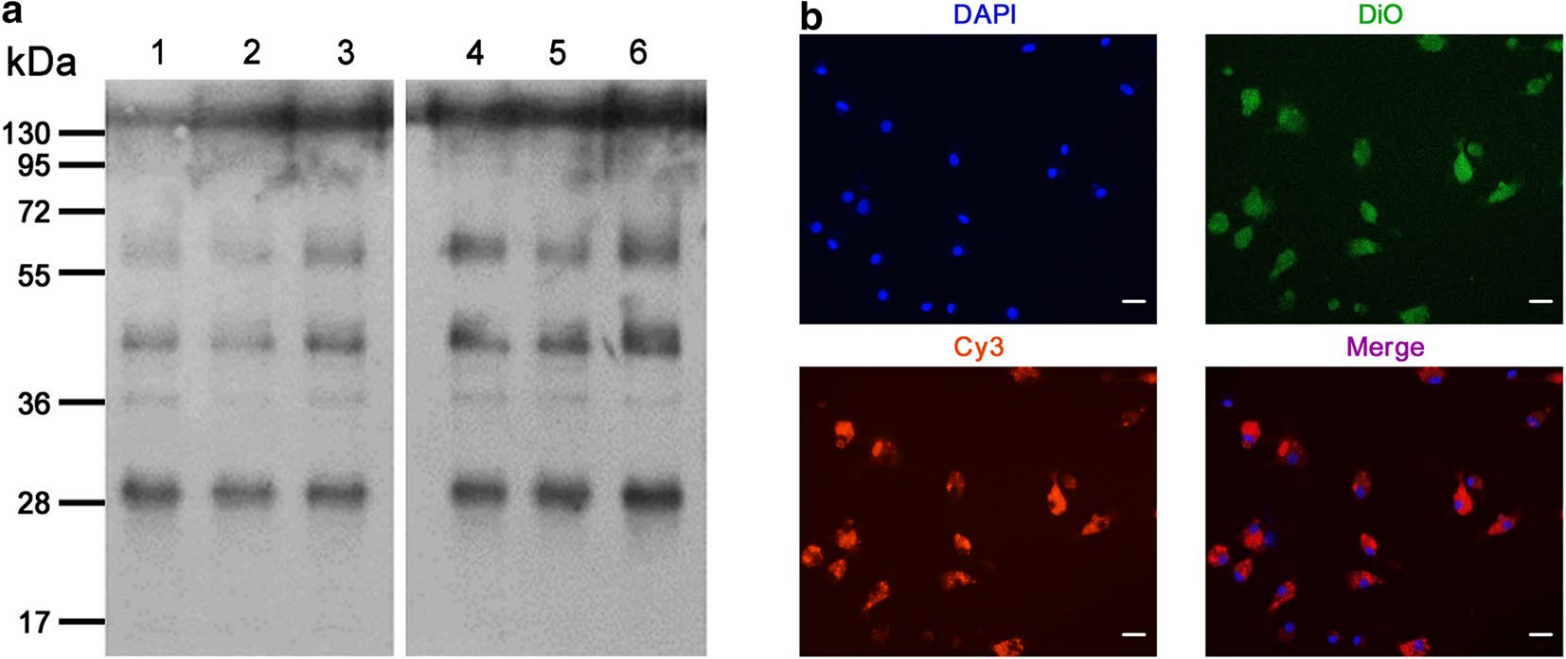
FgESPs do not directly interact with DNMT1 or TET1 in buffalo DCs. **a** Western blot analysis for the Co-IP assay to determine the interaction between FgESPs and DNMT1 or TET1 protein in buffalo DCs. Lanes 1–3: protein-G beads-eluted ‘Ag-Ab-unknown interactive protein’ complex protein sample specifically bounded to anti-DNMT1 mAb (Lane 1), anti-TET1 mAb (Lane 2) or normal mouse IgG (Lane 3) as control Ab from FgESPs-treated DCs; Lanes 4–6: protein-G beads-eluted ‘Ag-Ab-unknown interactive protein’ complex protein sample specifically bounded to anti-DNMT1 mAb (Lane 4), anti-TET1 mAb (Lane 5) or normal mouse IgG (Lane 6) from untreated DCs. The laboratory-made FgESPs-specific pAb was used as a primary antibody for all lanes. The immunoblotting result showed that the band pattern among all protein samples had no significant difference. **b** Fluorescent subcellular localization for FgESPs in buffalo DCs observed under a fluorescence inverted microscope. DAPI (blue) stained the cell nucleus; lipophilic dye DiO (green) strongly bound to the cell membrane; and Cy3-pre-labelled FgESPs emitted orange fluorescence. The merged image demonstrated that FgESPs might not locate in the nucleus. *Scale-bars*: 20 μm

To determine if there is a direct interaction between FgESPs components and DNMT1 or TET1 of buffalo DCs, we performed a reciprocal co-immunoprecipitation to test the bounding between FgESPs and either of DNMT1 or TET1 in DCs. Western blotting detection of protein complexes bound to anti-DNMT1 antibody or anti-TET1 antibody revealed no specific bands, either compared to cell samples from untreated DCs or to protein complexes bound to normal mouse IgG antibody (Fig. 4a), indicating that FgESPs did not form complex with either DNMT1 or TET1. Hence, we used some classic bioinformatics analytic tools to predict subcellular localization for buffalo DNMT1 and TET1 (Additional file 4: Figure S3). Phylogenetic analysis indicated that DNMT1 or TET1 of buffalo has high similarity (over 77.7%) with that of cattle, pig, human, monkey, gorilla and horse according to the multiple alignment of the amino-acid sequences. Prediction of TM domain using TMHMM showed that the predicted TM probabilities of the two targets are both ‘0’, suggesting that the TM domain is unlikely to exist in either of these two proteins. Prediction of SP domain showed that the C-, Y-, S- and mean-D-score of the amino-acid sequences of buffalo DNMT1 and TET1 were all below the default cut-off value, suggesting the absence of an SP domain in either of these two proteins. Both of the two buffalo proteins scored the highest inside the nucleus (ProtComp scoring: 9.0 and 8.9, respectively), and their nuclear localization probabilities (PSORT II prediction) reached 89.0% and 94.1%, respectively, indicating a high possibility to function in the nucleus. Then, we further performed a fluorescent subcellular localization for FgESPs in DCs, with the aim to confirm if FgESPs were not able to reach the cellular nucleus where DNMT1 or TET1 were mainly predicted to locate. Our result demonstrated that the Cy3-pre-labelled FgESPs (appearing orange) were seen located on the surface of or in the cytoplasm of the cells, but not distributed over any site of the cell nucleus (Fig. 4b). Together, these results suggest that FgESPs seem unlikely to bind or interact directly with DNMT1 or TET1 in buffalo DCs.

### The effect of FgESPs on DC development and functions may be indirectly related to DNMT1 or TET1

**Fig. 5:**
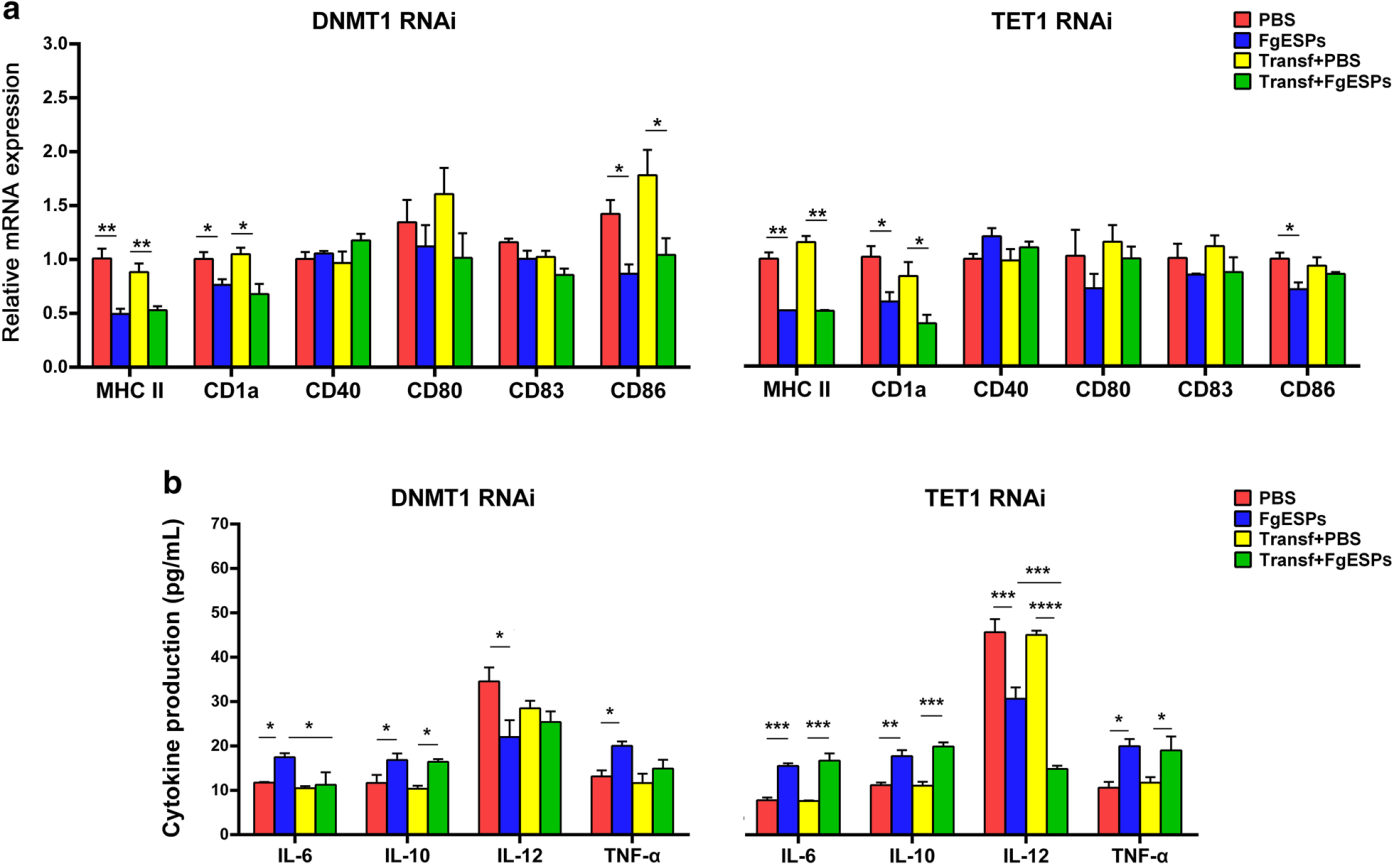
Effect of FgESPs on development and immune functions of buffalo DCs after DNMT1- or TET1-knockdown. **a** Transcriptional profile of DC development and immune function associated markers was determined by qRT-PCR. The data is represented as the relative mRNA expression level, calculated by 2^*−*ΔΔCq^ method. **b** Production of typical cytokines involved in buffalo DCs development and immune functions was measured by commercial ELISA kits. Columns in red and blue represent the normal DCs treated with PBS control (red) and FgESP (blue) alone, respectively, while the yellow and green columns represent the DNMT1 siRNA or TET1 siRNA-transfected DCs (with RNAi on corresponding targets) that were treated with the PBS control (yellow) and FgESP (green) alone, respectively. Representative histograms from three independent experiments are shown. All data are presented as the mean ± standard error (SE) of triplicate measurements. *P < 0.05, **P < 0.01, ***P < 0.001, ****P < 0.0001

Since we preliminarily inferred the lack of direct contact between FgESPs and DNMT1/TET1 in buffalo DCs, we assessed whether there was some hidden indirect interaction. As a critical premise and base, our preliminary test of comparative analyses on DC markers and cytokines in mock RNAi DCs and non-transfected DCs, has clearly shown that there was no significant difference between mock RNAi DCs and normal DCs (Additional file 5: Figure S4), suggesting that the current RNAi reagents or conditions would not introduce much change to the phenotype or cytokine production of buffalo primary DCs. The result of the following tests demonstrated that the siRNA interference of DNMT1/TET1 did not alter the transcriptional levels of all test DC markers, regardless of whether FgESPs were present or not (Fig. 5a). This indicates that knockdown of these two genes could not influence the DC phenotype or the regulatory pattern of FgESPs on DC development in vitro. Surprisingly, when comparing with FgESPs-treated normal buffalo DCs, the excretory level of IL-6 was significantly downregulated in DNMT1-knockdown DCs incubated with FgESPs (*F*_(3, 8)_ = 4.587, *P* = 0.0185), while the IL-12 content in the culture supernatant of TET1-knockdown DCs decreased over 50% (*F*_(3, 8)_ = 54.77, *P* = 0.0004) (Fig. 5b). Thus, we hypothesize a possibility that DNMT1 and TET1 are somehow involved in the regulatory mechanism of FgESPs to IL-6 and IL-12 production, respectively, in buffalo DCs.

## Discussion

Dendritic cells play an important role in the initiation and modification of helminth-specific immune responses, and are crucial to drive immunologic tolerance [14, 28]. The maturational state of mammal DCs could be altered to two completely contrary directions by the mixed ESPs or single ESP content from different parasite species, either promoting maturation [29], or suppressing maturation [8, 30]. FhESPs have been reported capable of impairing the ability of murine DCs being activated by TLR ligands and also their capacity to stimulate an allospecific response [9]. ESPs of *Trichinella spiralis* and *Clonorchis sinensis* could also induce tolerogenic properties in human and mouse DCs [31]. Similarly, as shown in our recent study, buffalo DCs activation mediated by FgESPs may appear to remain immature (or considered semi-mature), through promoting DNA methylation on some innate immunity-related pathways [19]. However, whether and how the FgESPs can influence the differentiation of buffalo DCs to further modulate the downstream differentiation of T cells, remains unclear.

Helminth parasites cause Th2 and Treg type immune responses but suppress the anti-parasitic Th1 response to maintain their longevity in the host through many efficient strategies [32, 33]. Studies have shown that some antigenic composition within parasite ESPs, like a filarial ES-62, has been found to redirect the transition of naïve DCs to the DC2 phenotype and induce a strong Th2 response [29, 34]. FhESPs-pre-treated mouse DCs were found to promote a polarization of Th cells to a bias of Th2 or Treg cells in MLR [4]. Within the FhESPs, glycoproteins could modulate the processes of antigen recognition, processing and presentation, which are considered to be involved in the activation of DCs, the tolerance of inflammation, and the induction of Th2 response; while CatL members could impair Th1 cytokine production then deactivate TLR signaling in DCs *via* blocking the effective ‘ligands-to-TLRs’ interaction [35]. Our present data showed that the FgESPs, like the function of FhESPs, may also potentially activate the DC2 phenotype of buffalo DCs and thereby direct Th2 cell differentiation in vitro, in accordance with most research results on helminth-secreted products [29, 34]. This result was also similar to our previous observation of a clear Th2-bias of the immune response in buffaloes which were chronically infected with *F. gigantica* [5, 18]. The potential of polarization towards a DC2 and Th2 response induced by FgESPs would possibly be due to the downregulation of Th1 cytokine and related pathway through MAPK/ERK phosphorylation that triggers the suppression of the NF-κB pathway downstream, like the findings for other helminths [36]. Further evidence has indicated that helminth ESPs may suppress DC1 polarization of DCs through TLR3/4 signaling [7, 35, 37]. As the involvement of TLR4 was also found in the infected buffalo liver [38], we therefore speculate that FgESPs may similarly induce a low-Th1/high-Th2 cytokine secretion in buffalo DCs to redirect the DCs toward DC2 type following the downstream Th2 polarization, possibly through disrupting TLR-associated antigen presentation. However, more data about TLR signaling pathways are still required to test this hypothesis.

To our surprise, when co-culturing buffalo DCs with homologous lymphocytes in our MLR test, the expression of all Treg markers, as well as the secretion of regulatory cytokines (IL-10 and TGF-β) increased, suggesting the possible presence of Treg differentiation. The ESPs of most helminth parasites, in general, induce cytokine production and effector cell polarization toward Th2/Treg type [39]. *Fasciola gigantica* may also take advantages of releasing ESPs to inhibit the pathogen damage and clearance of Th1 inflammation in the host immune cells during the infection, then creates a hypo-responsiveness of the host immune system. Interestingly, we noticed that the presence of FgESPs did cause a downregulation (rather than upregulation) of two DCreg markers (C1QA and STAB1) in the independent culture of DCs, which seemed to conflicted with the MLR results. FhESPs has been reported to induce tolerance of DCs [9]. The same study also suggested that tolerogenic DCs, which are partly produced by DC apoptosis [40, 41], exhibited a capacity to drive Th2 and Treg polarization of Th cells [9]. Therefore, we hypothesized that FgESPs may similarly mediate the apoptosis of buffalo DCs. The apoptotic DCs may further transformed into the tolerogenic DCs, which could promote the differentiation of T cells toward Th2/Treg cells without the increase of DCreg population. Our data indeed demonstrated that FgESPs alone could induce buffalo DCs to be in an early apoptosis state in vitro, characterized as typically apoptotic morphological changes, and significant alteration of the gene expression of apoptosis-associated molecular markers. It may therefore provide an explanation for such a strange mismatch of the DC2-tendency and the mixed Th2/Treg differentiation during co-culturing in the presence of FgESPs.

Tolerogenic DCs have the ability to upregulate the expression of IL-10 and TGF-β [42, 43]. It is notable that in our study, IL-10 was particularly found to be upregulated in FgESPs-treated DCs alone, as well as in the mixed cells of FgESPs-pre-treated DCs and lymphocytes, in both transcriptional and protein levels. IL-10, previously considered as a Th2-specific cytokine, is now accepted as an important multi-functional and tolerogenic cytokine involved in the generation and development of Treg cells [44]. IL-10 suppresses almost all functions of APCs in both innate and specific immunity, inhibits inflammatory cytokine production (mainly Th1) and T cell stimulatory capacities of APCs, and contributes to the immune tolerance during antigen persistence [44–46]. Also, IL-10 strongly enhances the spontaneous apoptosis of DCs in the lymphoid tissue [47]. IL-10 produced by DCs can limit or terminate ongoing inflammatory responses by inhibiting the proinflammatory cytokine production, through IL-10-mediated protein degradation [48]. In the buffalo infection model, IL-10 was also found to increase at both the mRNA level in the liver and the protein level in the serum. [5, 18]. So, the elevated IL-10 level found in FgESPs affected DCs alone or T cells co-cultured with DCs, may contribute to the suppression of Th1 cell differentiation, then the suppression of the Th1 response thereafter. The key regulatory function is possibly one of the key roles of FgESPs on limiting buffalo DC development and functions, which could be our next priority research point worthy of attention.

To lay a basic foundation for elucidating the mechanisms by which such FgESPs-activated DC2 phenotype influences Th2 and/or Treg cell development, we decided to delve further into the complex antigenic composition to investigate if there was component of FgESPs interacting with DCs through specific pathway(s) and to address the role of which in the regulation of Th1/Th2/Treg response. Since we previously have preliminarily discovered that DNA methylation of genes may play an important role in buffalo DCs [19], in the present study, we first measured the protein level of histone methylation, to determine the unsure relation between DNA methylation and histone methylation in buffalo DC development involved with FgESPs. Our data showed that FgESPs induced a slight enhancement of H3K9 methylation but a dramatic reduction of H3K4 methylation, while no significant change of the H3K27 methylation level was detected. The methylation states of H3K4, H3K9 and H3K27 are closely associated with the regulation of gene expression pattern during the transition of naïve DCs to active DCs or tolerance DCs [49]. Histone methylation of H3K4 lysine residual site actives transcription as an enhancer, while the methylation of H3K9 and H3K27 represses gene expression but serves unique functions [50–52]. Transcriptional repressive H3K9 and H3K27 methylations have a crucial role in regulating DNMT1-mediated DNA methylation recovery [53]. Herein, we confirmed the existence of FgESPs-induced methylation status changes in buffalo DCs at both DNA and histone levels, although it is still hard to explain the lack of alteration for H3K27 methylation without any data showing the demethylation status of major histones. In addition, we noticed a decreased expression of MHC II gene in FgESPs-treated DCs. Another study found that the MHC II expression increase sharply when the H3K9-specific histone methyltransferase was silenced [54]. Thus, we assume that FgESPs may probably influence the normal antigen presentation in buffalo DCs through disrupting the state of DNA and histone methylation, which is supported by the result of our previous methylated DNA immunoprecipitation sequencing (MeDIP-Seq) [19].

Based on this hypothesis, we selected DNMT1 and TET1, which are the most known and critical enzymes in regulating DNA methylation and hydroxymethylation, as two representative targets and initial pointcuts to further search functional parasite antigen(s) from FgESPs-DC interaction. We assumed that some functional antigen(s) of FgESPs could at least react with DNMT1 and/or TET1 in DCs. Given the very high similarities with most mammal species according to the amino-acid sequences, buffalo DNMT1 and TET1 are reasonably predicted to locate and function at the nucleus as the same as other reported species [55–58]. We thus would expect to see that FgESPs directly binds to the nucleus where DNMT1 and TET1 are located, and show specific binding reactions with DNMT1 and TET1. Unfortunately, neither the Co-IP assay nor the fluorescent subcellular co-localization assay had proven that FgESPs components could bind to or react with DNMT1/TET1 in buffalo DCs. In contrast, in our study, they did not even locate at the neighboring region in the cells after co-incubation for 24 h.

The failure of the tests for the direct reaction, made us alternatively turn to evaluate if there was any indirect reaction through DNA methylation-related elements and pathways. Interfering gene expression of DNMT1 or TET1 has been reported to alter the status of DNA methylation in mammal cells [22, 59]. Also, the DNMT1-dependent methylation or TET1-dependent hydroxymethylation would influence DC development and differentiation [60, 61]. Our strategy in this study was to knockdown the expression of DNMT1 or TET1 in buffalo DCs through RNAi technology [62], then to test if any significant influence would occur after FgESPs treatment. Even though interfering DNMT1/TET1 expression in mammal cells such as stem cells, could block the cell differentiation progress [63–65], there is no report of the reaction on ruminant DCs. In our study, we confirmed that the knockdown of both DNMT1 and TET1 gene expression in buffalo DCs were both successful in separate experiments. The result did show that the expression of DC markers has not been influenced so much after the knockdown of DNMT1 or TET1, which has not been described in any other ruminant DCs. Surprisingly, rather than non-RNAi normal DCs, the production of IL-6 and IL-12 in DNMT1-knockdown-DCs and TET1-knockdown-DCs, were found to separately alter when FgESPs were added to co-incubate with DCs. IL-6 is known to affect the differentiation of DCs and may shift the Th1/Th2 balance of T cells toward Th2 [66–68], whereas IL-12 also functions as a promotor for the differentiation and development of Th1 cells [69]. Our results here suggest that the presence of DNMT1 and TET1 determines the fate of the FgESPs-mediated DC development and differentiation, which may induce Th2 cell differentiation and inhibit the Th1 response, possibly through some undetermined pathways depending on these two proteins. Further researches into the interacting network between IL-6 and DNMT1, or between IL-12 and TET1 in buffalo DCs, and the distinct functions and mechanisms which account for the involvement of these genes and related pathways with the tolerogenic phenotype of DCs, would probably enlighten thoughts and concepts for the complex network and vague research direction of interaction between FgESPs and DCs.

## Conclusions

The present study investigated the in vitro effect of FgESPs in mediating the phenotypic change of buffalo DCs, as well as the downstream T cell differentiation which is highly orchestrated and regulated by distinct DC subset(s). We revealed that FgESPs may induce the DC2 phenotype and the apoptosis of buffalo DCs, and further induce the Th2/Treg differentiation of T cells. We therefore hypothesized that such an effect may be involved in the methylation processing under some certain manners relying on DNMT1- or TET1-related gene expression and pathway(s). Future research on these aspects may provide an explanation and more reasonable information. Our present study provides valuable information for better understanding the interactions between innate immune cells and the helminth parasite-derived molecules, and thus will boost the research and development of anti-fascioliasis therapies. Other genes and proteins that involve the progresses of DNA methylation/hydroxymethylation (for example, other DNMT and TET members) may also be investigated in our future studies for a broader and deeper understanding about the interaction between FgESPs and host immune cells.

## Supplementary information

**Additional file 1: Table S1.** List of primers used in the SYBR green-based qRT-PCR analysis.

**Additional file 2: Figure S1.** Preparation and identification of the laboratory-made rabbit polyclonal antibody (pAb) against whole antigen (Ag) of FgESPs. **a** Scheme of the procedure for generating FgESPs-specific pAb. **b** Characteristic band profile pattern of FgESPs shown by SDS-PAGE following Coomassie blue staining; bovine serum albumin (BSA) served as a standard control. **c** Western-blot assay confirmed the specific binding of the generated pAb to FgESPs. Lane M: Protein molecular weight marker; Lane 1: 10 μl of BSA (5 μg, in PBS); Lane 2: 10 μl of FgESPs (20 μg, in PBS); Lane 3: 10 μl of FgESPs (40 ng, in PBS).

**Additional file 3: Figure S2.** Knockdown of DNMT1 and TET1 genes using siRNA in buffalo DCs. **a** Relative mRNA expression of DNMT1 or TET1 separated following siRNA transfection in generated buffalo DCs determined by qRT-PCR. Representative histograms from three independent experiments are shown. ** *P* < 0.01. **b** The expression of DNMT1 and TET1 in buffalo DCs following RNAi measured by western blotting (upper) and grayscale analysis (lower). *** *P* < 0.001.

**Additional file 4: Figure S3.** Prediction of protein subcellular localization for buffalo DNMT1 and TET1. **a** Phylogenetic analysis of DNMT1 and TET1 among common mammal host species based on the multiple alignment of the amino-acid sequences. **b** Prediction of protein transmembrane (TM) domain by using TMHMM Serve v2.0 online software. **c** Prediction of signal peptide (SP) domain by using SignalP v4.1.

**Additional file 5: Figure S4.** Expression of DC markers (**a**) and production of cytokines (**b**) in non-transfected buffalo DCs and DCs that were transfected with mock siRNA for DNMT1 (left) or TET1 (right). Representative histograms from two independent experiments are shown. * *P* < 0.05, ** *P* < 0.01, *** *P* < 0.001.

## Data Availability

The datasets supporting the findings of this article are included within the article and its additional files.

## Abbreviations

APC: antigen-presenting cell
BCA: bicinchoninic acid
C1QA: complement component 1 q subcomponent A chain
CD: cluster of differentiation
Co-IP: co-immunoprecipitation
Cq: quantification cycle
DC: dendritic cell
DNMT: DNA methyltransferase
ELISA: enzyme-linked immunosorbent assay
FgESPs: *Fasciola gigantica* excretory/secretory products
GAPDH: glyceraldehyde-3-phosphate dehydrogenase
GATA3: GATA binding protein 3
H3K4: lys4 of histone H3
IFN-γ: interferon gamma
IL: interleukin
MeDIP-Seq: methylated DNA immunoprecipitation sequencing
MHC: major histocompatibility complex
MLR: mixed lymphocyte reaction
NCBI: National Center for Biotechnology Information
PBMC: peripheral blood mononuclear cell
PBS: phosphate-buffered solution
PFA: paraformaldehyde
qRT-PCR: quantitative reverse transcriptase real-time polymerase chain reaction
RNAi: RNA interference
siRNA: small interfering RNA
STAB1: stabilin-1
TEM: transmission electron microscopy
TET: ten-eleven translocation
TGF-β: transforming growth factor beta
TLR: toll-like receptor
TNF-α: tumor necrosis factor alpha
