# Supplementary material for: Fasciola gigantica excretory-secretory products (FgESPs) modulate the differentiation and immune functions of buffalo dendritic cells through a mechanism involving DNMT1 and TET1
Source: Parasit Vectors. 2020 Jul 17;13:355. doi: 10.1186/s13071-020-04220-0 (PMC7368760; doi:10.1186/s13071-020-04220-0)
Supplement: Supplementary file 1 — Additional file 1: Table S1. List of primers used in the SYBR green-based qRT-PCR analysis. [file 13071_2020_4220_MOESM1_ESM.docx]

**Additional file 1: Table S1** List of primers used in the SYBR green-based qRT-PCR analysis

| **Gene** | **Primer sequence (5' to 3')** | **Gene** | **Primer sequence (5' to 3')** |
| --- | --- | --- | --- |
| **IFN-γ**^[1]^ |  | **Bcl-2**^[7]^ |  |
| Forward: | GTCTCCTTCTACTTCAAACT | Forward: | TCAATTGTCGTGGCATCAAAA |
| Reverse: | ATTCTGACTTCTCTTCCGCT | Reverse: | CCCCCGACACCTGTTAGCTT |
| **IL-4**^[2]^ |  | **Mcl-1** |  |
| Forward: | CAGCATGGAGCTGCCT | Forward: | GGCAGGATTGTGACTCTTATTT |
| Reverse: | ACAGAACAGGTCTTGCTTGC | Reverse: | TCCCAGCCTCTTTGTTTGA |
| **IL-10**^[3]^ |  | **CD1a**^[8]^ |  |
| Forward: | CTGTGCCTCTCCCCTAGAGT | Forward: | TGTGCCACGTCTCAGGATT |
| Reverse: | GCAGCTAGCTCCACAAGGAA | Reverse: | TGTCCTGGTCTCCTAGACTGC |
| **IL-13**^[4]^ |  | **CD40**^[9]^ |  |
| Forward: | AGAACCAGAAGGTGCCGCT | Forward: | GGGCTTTTGGATACCGTCTGT |
| Reverse: | GGTTGAGGCTCCACACCATG | Reverse: | AGCAGATGACACGTTGGAGAAG |
| **C1QA** |  | **CD80**^[9]^ |  |
| Forward: | CCCAGGCTACTACTATTTCACC | Forward: | CCATCCTGCCTGGAAAAGTG |
| Reverse: | GAAAGAATCCCTTGCTGTTG | Reverse: | GGTTATCGTTCATGTCAGTGATGGT |
| **STAB1** |  | **CD83**^[10]^ |  |
| Forward: | ATGTCCTCCTGACTATGGG | Forward: | TGTGAAGCCCTTTGCTTGAC |
| Reverse: | GGCTGTGACTTGGCTGT | Reverse: | CCAAGAGGTTGACCAGATAG |
| **T-bet**^[5]^ |  | **CD86**^[11]^ |  |
| Forward: | CCTGGACCCAACTGTCAACT | Forward: | AGAAGGTCCCAAGGACTGGT |
| Reverse: | GAAACTCGGCCTCATAGCTG | Reverse: | GCTTGGCACAGGTGACTTTG |
| **GATA3**^[6]^ |  | **MHC II**^[12]^ |  |
| Forward: | GATCAAGCCCAAGCGAAGG | Forward: | CCTCGCTTGCCTGAATTTGC |
| Reverse: | CCGCAGGCATTGCAGACA | Reverse: | ACAGGTGCCGACTGATGC |
| **Foxp3**^[5]^ |  | **DNMT1** |  |
| Forward: | GACAGCACCCTTTCGACTGT | Forward: | ACCCTGACTCCACCTACGA |
| Reverse: | CTCCAGAGATTGCACCACCT | Reverse: | ACTTGCTCCACCACGAACT |
| **TFG-β**^[6]^ |  | **TET1** |  |
| Forward: | CGTGCTAATGGTGGAATAC | Forward: | ACTAAAGCCTCCGAGCAGA |
| Reverse: | GCCAGGAATTGTTGCTATA | Reverse: | GAAAAGCCTTCAGACCCAAT |
| **Bax**^[7]^ |  | **GAPDH**^[13]^ |  |
| Forward: | TTTCTGACGGCAACTTCAAC | Forward: | ACGTGTCTGTTGTGGATCTGAC |
| Reverse: | GGGTGTCCCAAAGTAGGAGAG | Reverse: | CGCTGTTGAAGTCGCAGGAG |
|  |  |  |  |

**References**

1. Mingala CN, Konnai S, Venturina FA, OnumaM, Ohashi K. Quantification of water buffalo (Bubalus bubalis) cytokine expression in response to inactivated foot-and-mouth disease (FMD) vaccine. Res Vet Sci. 2009;87:213–7.

2. Puech C, Dedieu L, Chantal I, Rodrigues V. Design and evaluation of a unique SYBR Green real-time RT-PCR assay for quantification of five major cytokines in cattle, sheep and goats. BMC Vet Res. 2015;11:65.

3. Castillo-Velázquez U, Aranday-Cortés E, Gutiérrez-Pabello JA. Alternative activation modifies macrophage resistance to *Mycobacterium bovis*. Vet Microbiol. 2011;151:51–9.

4. Piper EK, Jackson LA, Bagnall NH, Kongsuwan KK, Lew AE, Jonsson NN. Gene expression in the skin of Bos taurus and Bos indicus cattle infested with the cattle tick, *Rhipicephalus* (*Boophilus*) *microplus*. Vet Immunol Immunop. 2008;126:110–9.

5. Maeda Y, Ohtsuka H, Tomioka M, Oikawa M. Effect of progesterone on Th1/Th2/Th17 and regulatory T cell-related genes in peripheral blood mononuclear cells during pregnancy in cows. Vet Res Commun. 2013;37:43–9.

6. Shi W, Wei ZY, Elsheikha HM, Zhang FK, Sheng ZA, Lu KJ, et al. Dynamic expression of cytokine and transcription factor genes during experimental *Fasciola gigantica* infection in buffaloes. Parasite Vector. 2017;10:602.

7. Zhao XM, Hao HS, Du WH, Zhao SJ, Wang HY, Wang N, et al. Melatonin inhibits apoptosis and improves the developmental potential of vitrified bovine oocytes. J Pineal Res. 2016;60:132–41.

8. Pleasance J, Wiedosari E, Raadsma HW, Meeusen E, Piedrafita D. Resistance to liver fluke infection in the natural sheep host is correlated with a type‐1 cytokine response. Parasite Immunol. 2011;33:495–505.

9. Lei LY, Hostetter JM. Limited phenotypic and functional maturation of bovine monocyte-derived dendritic cells following Mycobacterium avium subspecies paratuberculosis infection *in vitro*. Vet Immunol Immunop. 2007;120:177–86.

10. Dego OK, Oliver S, Almeida R. Host–pathogen gene expression profiles during infection of primary bovine mammary epithelial cells with *Escherichia coli* strains associated with acute or persistent bovine mastitis. Vet Microbiol. 2012;155:291–7.

11. Mauro A, Russo V, Marcantonio L, Berardinelli P, Martelli A, Muttini A, et al. M1 and M2 macrophage recruitment during tendon regeneration induced by amniotic epithelial cell allotransplantation in ovine. Res Vet Sci. 2016;105: 92-102.

12. Lange-Consiglio A, Perrini C, Bertero A, Esposti P, Cremonesi F, Vincenti L. Isolation, molecular characterization, and *in vitro* differentiation of bovine Wharton jelly-derived multipotent mesenchymal cells. Theriogenology. 2017;89:338–47.

13. Kumar P, Yadav P, Verma A, Singh D, De S, Datta TK. Identification of stable reference genes for gene expression studies using quantitative real time PCR in buffalo oocytes and embryos. Reprod Domest Anim. 2012;47:88–91.
