# Supplementary figures and images for: Fasciola gigantica excretory-secretory products (FgESPs) modulate the differentiation and immune functions of buffalo dendritic cells through a mechanism involving DNMT1 and TET1
Source: Parasit Vectors. 2020 Jul 17;13:355. doi: 10.1186/s13071-020-04220-0 (PMC7368760; doi:10.1186/s13071-020-04220-0)

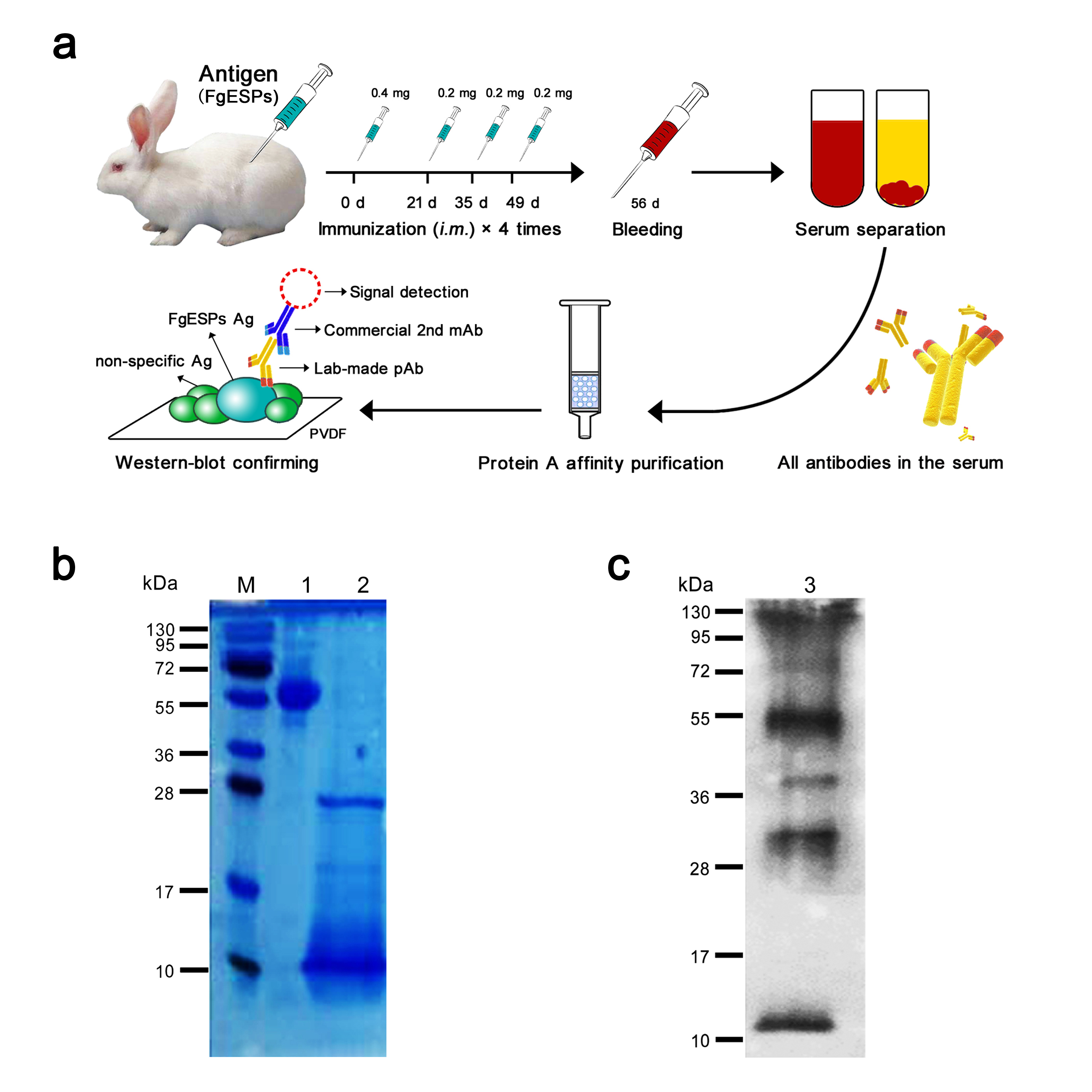

Supplement: Supplementary file 2 — Additional file 2: Figure S1. Preparation and identification of the laboratory-made rabbit polyclonal antibody (pAb) against whole antigen (Ag) of FgESPs. a Scheme of the procedure for generating FgESPs-specific pAb. b Characteristic band profile pattern of FgESPs shown by SDS-PAGE following Coomassie blue staining; bovine serum albumin (BSA) served as a standard control. c Western-blot assay confirmed the specific binding of the generated pAb to FgESPs. Lane M: Protein molecular weight marker; Lane 1: 10 μl of BSA (5 μg, in PBS); Lane 2: 10 μl of FgESPs (20 μg, in PBS); Lane 3: 10 μl of FgESPs (40 ng, in PBS). [file 13071_2020_4220_MOESM2_ESM.jpg]

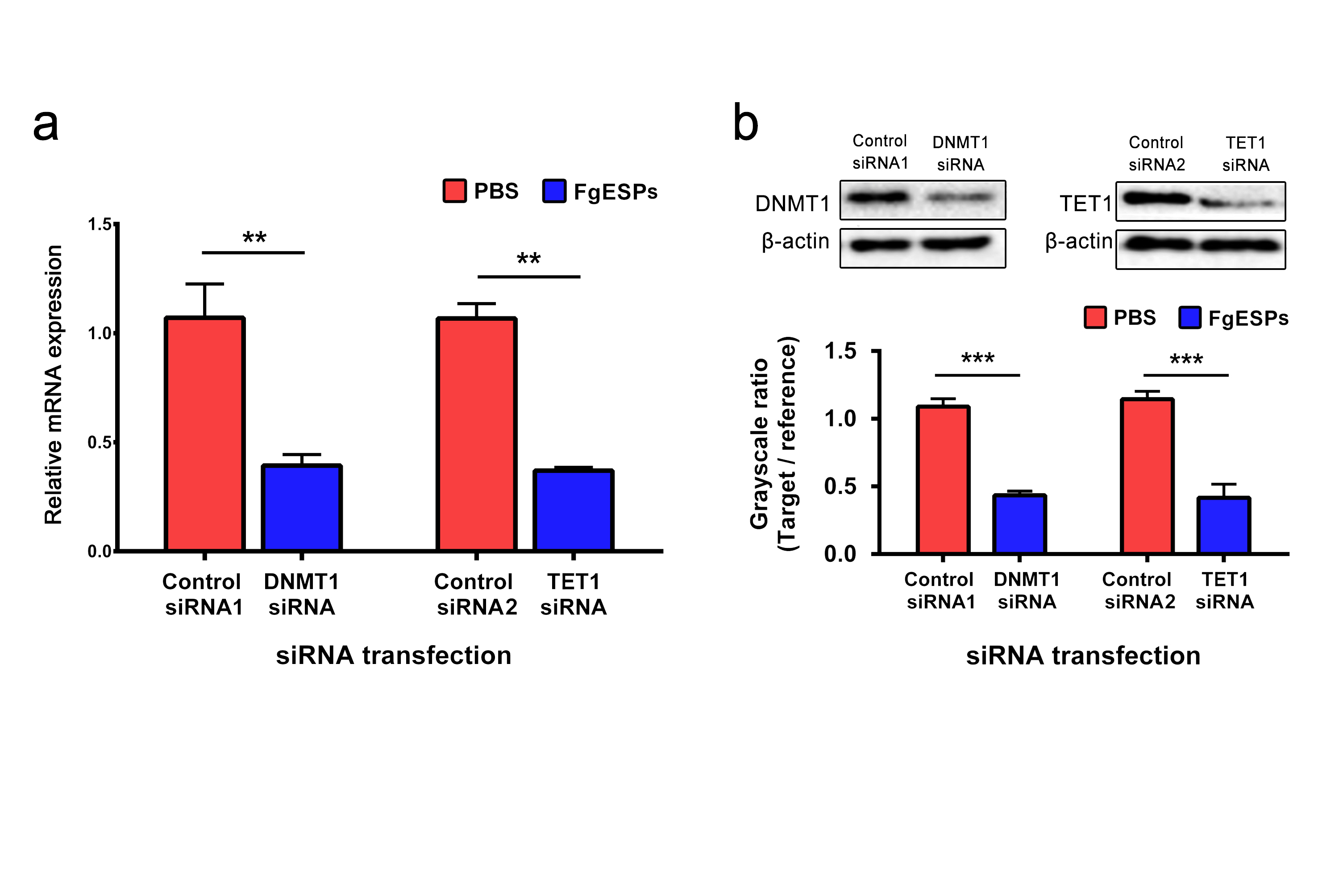

Supplement: Supplementary file 3 — Additional file 3: Figure S2. Knockdown of DNMT1 and TET1 genes using siRNA in buffalo DCs. a Relative mRNA expression of DNMT1 or TET1 separated following siRNA transfection in generated buffalo DCs determined by qRT-PCR. Representative histograms from three independent experiments are shown. ** P < 0.01. b The expression of DNMT1 and TET1 in buffalo DCs following RNAi measured by western blotting (upper) and grayscale analysis (lower). *** P < 0.001. [file 13071_2020_4220_MOESM3_ESM.jpg]

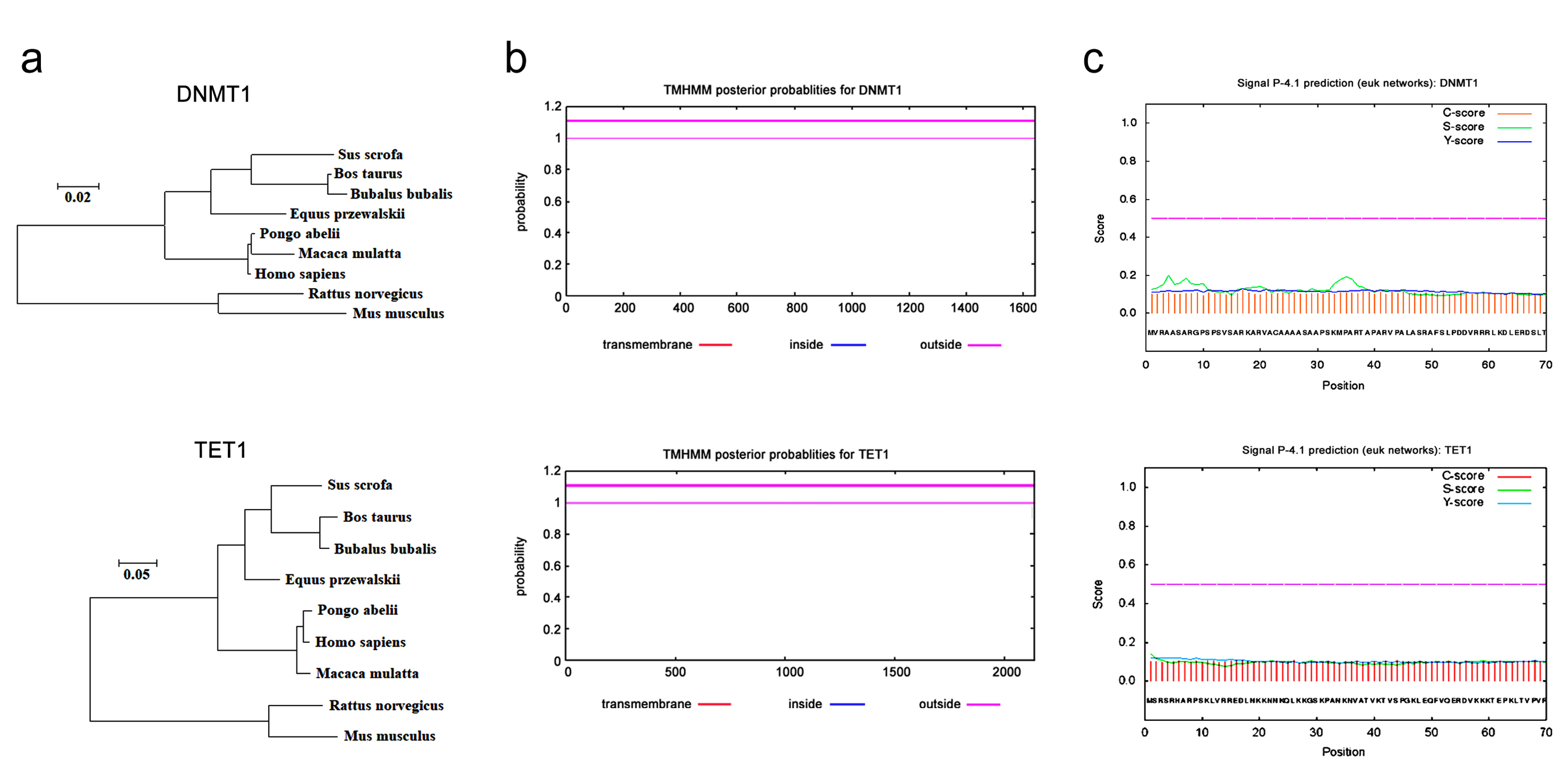

Supplement: Supplementary file 4 — Additional file 4: Figure S3. Prediction of protein subcellular localization for buffalo DNMT1 and TET1. a Phylogenetic analysis of DNMT1 and TET1 among common mammal host species based on the multiple alignment of the amino-acid sequences. b Prediction of protein transmembrane (TM) domain by using TMHMM Serve v2.0 online software. c Prediction of signal peptide (SP) domain by using SignalP v4.1. [file 13071_2020_4220_MOESM4_ESM.jpg]

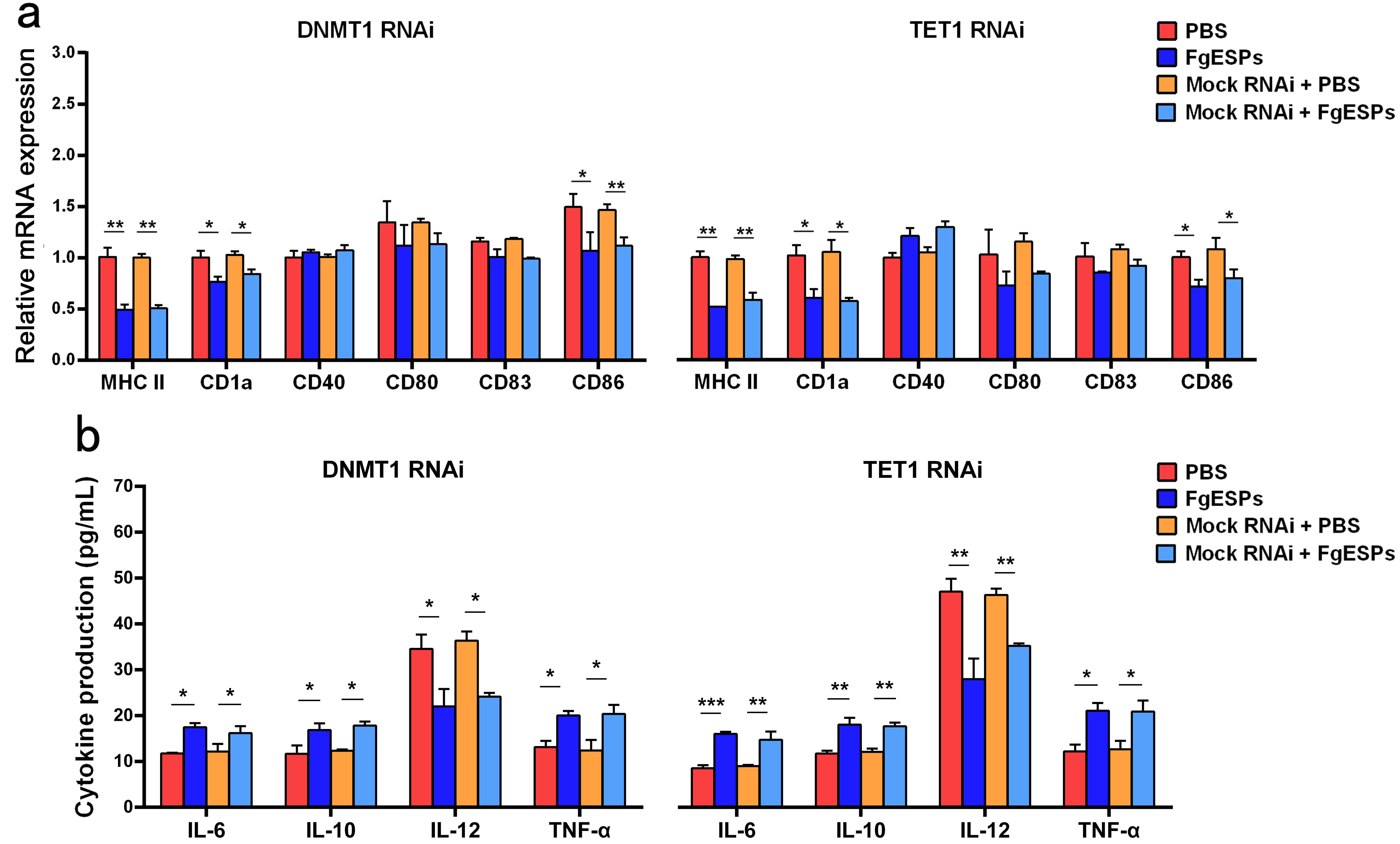

Supplement: Supplementary file 5 — Additional file 5: Figure S4. Expression of DC markers (a) and production of cytokines (b) in non-transfected buffalo DCs and DCs that were transfected with mock siRNA for DNMT1 (left) or TET1 (right). Representative histograms from two independent experiments are shown. * P < 0.05, ** P < 0.01, *** P < 0.001. [file 13071_2020_4220_MOESM5_ESM.jpg]
